# Low-Profile and Wider-Angle Beam Tilting Parasitic Array Resonator Antenna with Optimized Deflected Ground Plane on FR-4 Substrate

**DOI:** 10.3390/mi14040834

**Published:** 2023-04-11

**Authors:** Nur Ain Fatihah Mohd Zainudin, Mohamed Nasrun Osman, Thennarasan Sabapathy, Muzammil Jusoh, Mohd Najib Mohd Yasin, Mohamad Kamal A. Rahim

**Affiliations:** 1 Faculty of Electronic Engineering & Technology, UniMAP Pauh Putra Main Campus, Universiti Malaysia Perlis (UniMAP), Arau 02600, Malaysia; 2 Advanced Communication Engineering (ACE) Centre of Excellence, Universiti Malaysia Perlis, No 15 & 17, Jalan Tiga, Pengkalan Jaya Business Centre, Kangar 01000, Malaysia; 3 Advanced RF & Microwave Research Group, Faculty of Electrical Engineering, Universiti Teknologi Malaysia (UTMJB), Johor Bahru 81310, Malaysia

**Keywords:** beam tilting, pattern reconfigurable antenna, parasitic patch, deflected ground plane, RF switches

## Abstract

A low-profile and wide-angle radiation pattern reconfigurable antenna is designed, analyzed, and fabricated for wireless sensor network (WSN) applications, which operate at a 2.5-GHz frequency. This work aims to minimize the number of switches and optimize the parasitic size and ground plane to achieve a steering angle of more than 30° using a low cost-high loss FR-4 substrate. The radiation pattern reconfigurability is achieved by introducing four parasitic elements surrounding a driven element. In this work, the single driven element is fed by a coaxial feed, while other parasitic elements are integrated with the RF switches on the FR-4 as the substrate with dimensions of 150 × 100 mm (1.67 × 2.5 λ_o_). The RF switches of the parasitic elements are surface mounted on the substrate. By truncating and modifying the ground plane, the beam steering can be achieved at more than 30° on the xz plane. Additionally, the proposed antenna can attain an average tilt angle of more than 10° on the yz plane. The antenna is also capable of attaining other important results, such as a fractional bandwidth of 4% at 2.5 GHz and an average gain of 2.3 dBi for all configurations. By adopting the ON/OFF condition on the embedded RF switches, the beam steering can be controlled at a certain angle, thus increasing the tilting angle of the wireless sensor networks. With such a good performance, the proposed antenna has high potential to serve as a base station in WSN applications.

## 1. Introduction

Recent wireless sensor networks (WSNs) greatly demand high energy-efficient networks. To achieve this goal, there are a need and requirement to have wireless components capable of sending signals more efficiently to reduce energy waste. Such a capability is attainable with the solution of a radiation pattern reconfigurable (RPR) antenna [1]. Due to inflexible qualities, conventional antennas in WSNs may have limitations in meeting requirements and adapting to new conditions. On the other hand, the RPR antenna is capable of overcoming this issue with its ability to steer the main beam toward the desired direction. The alternative to the RPR antenna is known as beamforming, in which an array of the antenna can be used to steer it in such a way that the interfering signal from a particular direction can be canceled while improving the signal from the desired direction [2,3].

WSN sensors normally connect with a local hub in a single-hop manner like conventional WSNs; however, in later generations of WSNs, certain sensors can function as relays to communicate with each other in the multi-hop fashion as a form of cooperation. These multi-hop WSNs can be used to expand traditional WSNs to new applications, such as emergency services that require more flexibility in their activities. Using an RPR antenna can improve the beam steering of the WSN better than the directional antenna as RPRs can focus on improving the angle tilting of the beam steering of the antenna to a certain direction, while directional antennas are more focused on data reliability and the area of coverage [4].

The key limitation that can be found with the RPR antenna is the use of more RF switches to achieve multiple beam directions and beam steering capability. Apart from this limitation, the tilt angle of the steered beam is expected to be severely disrupted with the use of high-loss substrates, such as FR4. However, a low-cost design with a smaller number of switches is required for WSN applications. The RPR antenna could be a good candidate for WSN antennas that need to be able to direct beams. Nevertheless, several studies have indicated that RPR antennas require additional switching to conduct multiple-direction radiation pattern reconfiguration [5]. This need is a concern because more switches would use more electricity. It is necessary to create an RPR antenna with the fewest possible switches and multiple beam directions. The use of an RPR antenna with the largest number of switches and components reduced the antenna’s beam steering performance. Currently, the highest tilt angle that a three-element RPR antenna can achieve is usually around ±30° [6]. As is generally known, it should be emphasized that the proposed antenna was developed utilizing a Taconic substrate, which has low loss.

References [7,8,9] proposed a beam steering configuration and managed to achieve a good steering angle using Rogers substrate. The latter even required 12 switches to achieve that performance. Another complex structure using Rodgers has been presented in [10,11]. However, the issue of this work is the use of a complicated structure and multilayers with the use of a large number of switches, making it unsuitable for energy-efficient applications. Multiple direction-beam parasitic-based antennas have also been presented in [12], using Taconic substrate to achieve beam tilting of a maximum of a 25° angle. Work performed in [13,14] attempted to design a radiation pattern reconfigurable on FR-4 substrate using a PIN diode and circular-shaped structure. However, the work is only capable of achieving a maximum tilting angle of 15° and a maximum of 26°, respectively. Another paper [15] proposed FR-4 and integrated an electromagnetic bandgap also obtained a maximum angle tilting of ±26°. More than a 30° tilting angle has been achieved in [16,17] using an FR-4 substrate. However, these works required more complex techniques, such as metamaterials and extra switches. In addition, it is only capable of steering in the left and right directions. The pattern’s reconfigurability has also been accomplished by inserting the RF switches on the feeding network by modifying the current flow on it [18,19]. Although this method managed to achieve multi-directional beam steering capability, it required a complex structure, particularly to maintain the impedance and the matching of the feeding.

If a high loss substrate such as FR-4 is utilized, the tilting angle is expected to be reduced. As a result, it is sensible to achieve a performance of the angle tilting equal to or greater than 30° using a low-cost and high-loss substrate. The parasitic elements are added to the RPR antenna to achieve better gain. The parasitic also can act as a reflector to steer the beam to the desired angle. Thus, by adding the parasitic to the RPR antenna, the power consumed to radiate the signal also can be reduced. To obtain more beam directions with good gain but with fewer switches would be a challenging factor in designing an RPR antenna. In this work, the use of the RPR antenna is specifically dedicated to the WSN application; therefore, the number of switches should be fewer than six to avoid high power consumption, while the designed RPR antenna should minimum maintain beam steering angles, which should be at least in three directions.

Thus, in this work, the RPR antenna is chosen to improve the angle tilting of the beam steering of the antenna for WSNs, and a rectangular patch with parasitic elements is added to the FR-4 substrate. The switching components are situated on the different planes of the parasitic elements, and the coaxial probe feed excites the primary radiator, which is likewise located in the middle of the structure. This work focuses on developing multiple beam directions for an antenna with angle tilting of more than 30° using a high-loss substrate and fewer switches.

## 2. Antenna Design, Approach, and Configurations

A initial microstrip patch antenna is designed with four elements of the parasitic on the substrate with a full ground. The analysis begins with a full ground plane first and uses an artificial switch (ideal diode) for proof of the concept. Then, the realization of the RF switches on the design is further verified once the optimization with ideal diodes is completed.

**Figure 1:**
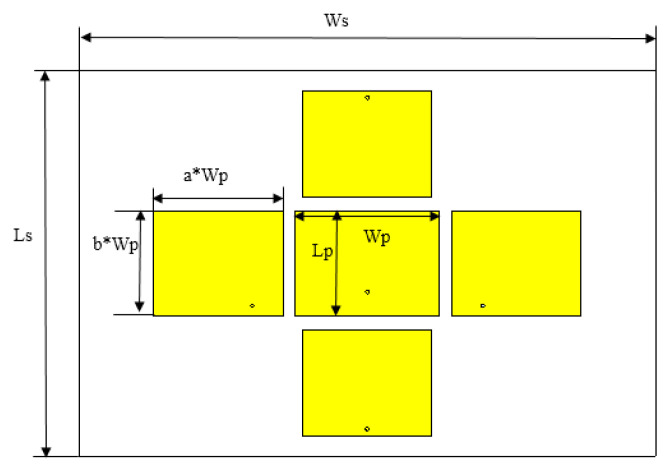
The geometry of the proposed structure.

Figure 1 shows the geometry of the structure. The dimension of the antenna’s FR-4 substrate is *L_s_* × *W_s_* (150 × 100 mm, 1.67 × 2.5 λ_o_), with height of 1.6 mm and permittivity of *ε_r_* = 4.3. The patch of the driven element (*L_p_* × *W_p_*) and all the parasitic elements are placed on the top of the substrate surface surrounding the driven element with the slightest different in size from the driven element, as shown in Figure 1. This choice is made because, when the size of the driven and parasitic elements is almost identical, the mutual coupling becomes stronger. On the other side of the substrate is the full ground with the same dimension as the substrate. The material used for the patch and the ground plane is copper with a thickness of *t* = 0.035 mm.

In each parasitic element, artificial switches via shorting pins are connected to achieve the reconfigurable beam antenna. The main radiator positioned in the middle of the structure is excited by the coaxial probe feed. The technique proposed here is a parasitic pattern array based on the Yagi–Uda concept. The parasitic patch is designed to surround the driven patch at the center. By controlling the switch whether the parasitic element is grounded or not, the behavior of the parasitic element can act as a reflector or director. The parasitic becomes a reflector when the artificial switch is active and connected to the ground, known as the ON state. In contrast, the parasitic becomes a director when the artificial switch does not activate and is not connected to the ground, known as the OFF state. The artificial switches are positioned at the specific location on the parasitic patch, and the direction of the beam steering is changed to the left, down and upward, and downward depending on the switching configuration, as tabulated in Table 1.

**Figure 2:**
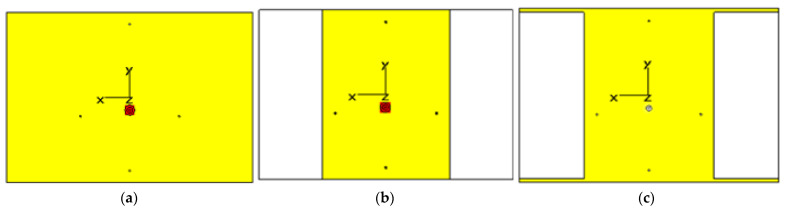
Ground plane optimization step: (**a**) full ground plane; (**b**) DGP; and (**c**) optimized DGP.

**Figure 3:**
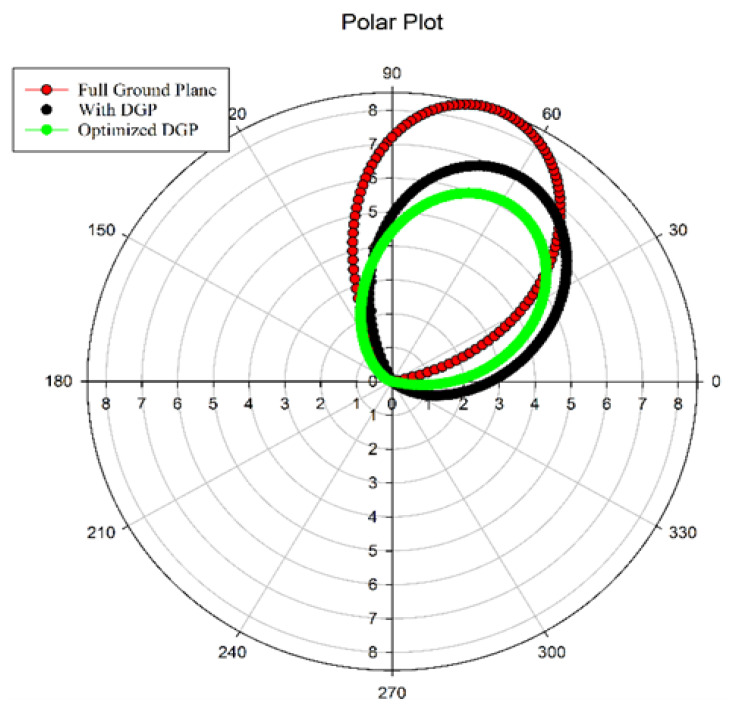
The polar plot radiation pattern for a different type of ground plane.

One important structure that plays a key role in determining the beam steering of the antenna is the ground plane. The length and width of the ground greatly affect the steering angle. The optimization step has been undertaken through parametric study to obtain a good steering angle for the antenna. The step of optimization is depicted in Figure 2. Based on the study, by truncating the ground plane to create a deflected ground plane (DGP), the tilt angle can be improved. The ground is first cut into 1/3, 1/2, and 3/4 of the original size. The optimized DGP is obtained by cutting the ground plane by half of the original parameters of the ground plane from the antenna design, and the steering angle is improved from 19 to 30°. From the data of the antenna design with the DGP, the antenna is optimized again until the maximum angle tilt is obtained. This tilt is accomplished by adding a ground on both sides of the antenna without touching the left and right patches. By adding a ground on both sides at the bottom and top, the tilt angle is further improved. The result of the different forms of ground planes toward tilting angles is shown in Figure 3. Table 2 summarizes the effect of the DGP on the tilting angle for all configurations. The DGP technique has improved the tilt angle results for the left and right configurations, while it has reduced the tilt angle for configurations III and IV. This outcome is the result of the truncated ground and reduces the reflection strength of the top and bottom parasitics. The main aim of this work is to improve the tilt angle of configurations I and II. Therefore, the optimization of the DGP is conducted in the design, as the degree of the angle tilting is increased for left-right configurations while still maintaining a good trade-off between the gain and the tilt angles of the remaining configurations.

**Figure 4:**
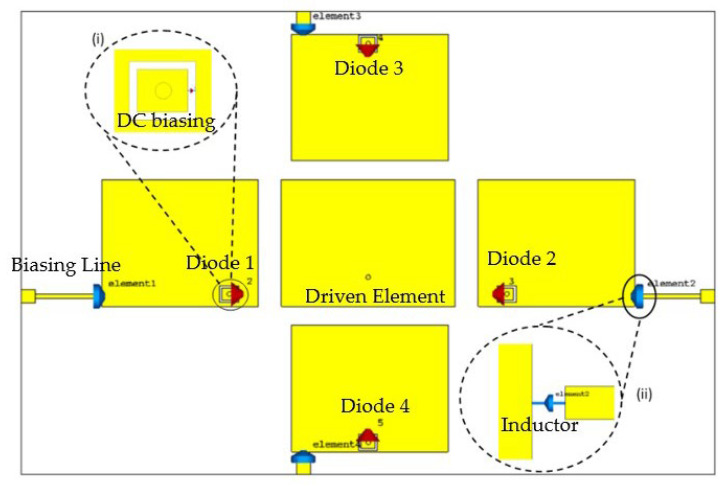
Front view of the optimized design of four-element parasitic with integration of biasing network for RF switches.

**Figure 5:**
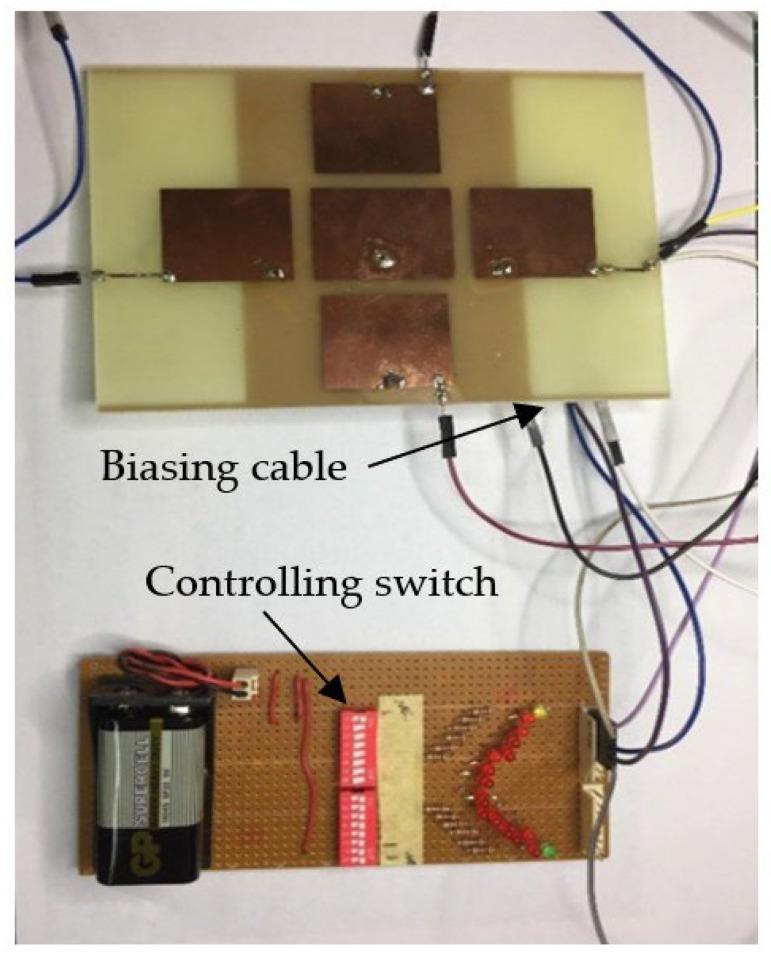
Fabricated antenna prototype with external switching board.

Figure 4 illustrates the antenna structure accommodated with DC biasing line and RF components, such as PIN diodes and inductors. The notation (i) and (ii) in the figure body shows the zoom area of (i) pin diode biasing and (ii) position of the inductor. To complete the biasing network, a minor adjustment is made to the design. A rectangular-shaped narrow slot with a width of 0.3 mm is designed to locate the PIN diode and separate it between the anode and cathode terminals (isolate DC biasing). This polarity of the PIN diode determines the positive and negative areas of the DC voltage supply for biasing purposes. Four RF choke inductors, with a value of 27 nH, are positioned between the parasitic patch and biasing line to improve the isolation between the RF current and the DC supply [20]. The anodes of the diodes are connected in small rectangles and linked to the ground via shorting copper. Meanwhile, the cathodes are connected to the remaining area of the parasitic patch via biasing line. For the activation of the diodes, the external switching control board is constructed. The switching board is composed of copper wire, a 9-V DC battery, a dip switch to control the biasing, and resistors for current limitation. Resistors with a value of 100 Ω are chosen so that a forward biased current of 90 mA is obtained (ON-state) and a reverse biased angle in the OFF-state condition. A photograph of the fabricated antenna is shown in Figure 5.

## 3. Results and Discussion

**Figure 6:**
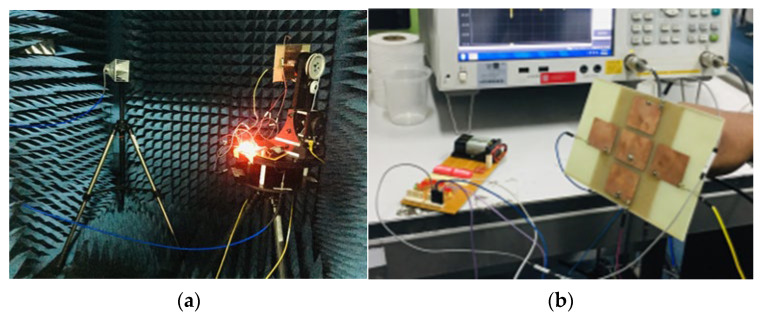
Measurement setup for (**a**) reflection coefficient and (**b**) radiation pattern.

The fabricated antenna is measured in terms of S-parameters and radiation patterns. The measurement is performed using the network analyzer for the frequency range of 1 GHz to 3 GHz. The far-field radiation pattern is measured at the resonant frequency on the E-plane and H-plane using the anechoic chamber at Advanced Communication Engineering (ACE), Universiti Malaysia Perlis. The measurement setup is displayed in Figure 6.

**Figure 7:**
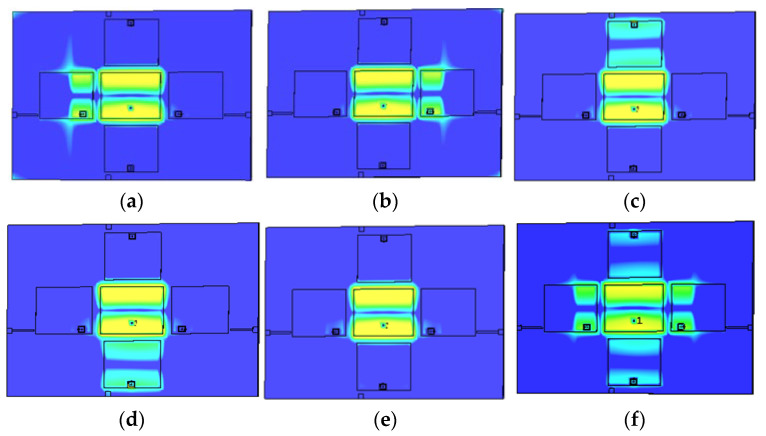
Simulated surface current distribution at 2.5 GHz: (**a**) left; (**b**) right; (**c**) top; (**d**) bottom; (**e**) all OFF; and (**f**) all ON.

Figure 7 shows the simulated current distribution at 2.5 GHz. As mentioned previously, one of the crucial factors that needs to be considered is the mutual coupling effect, electromagnetic reactions that exist between driven and parasitic elements. As can be seen in Figure 7, for the left configuration, in which the PIN diode 1 is OFF, more current has been coupled to the left parasitic, and the parasitic has become the directive for radiation to tilt the beam, while the other parasitics act as reflectors to push away the beam toward the directive parasitic. Similar behavior can be seen for the configurations at the right, top, and bottom. As expected, for all ON and OFF configurations, there is no tilting angle due to no parasitic being grounded to act as a director to pull the beam. Even though there is no beam tilting, the gain for all OFF drops to 1.41 dBi due to all parasitics having been grounded and acting as directors.

**Figure 8:**
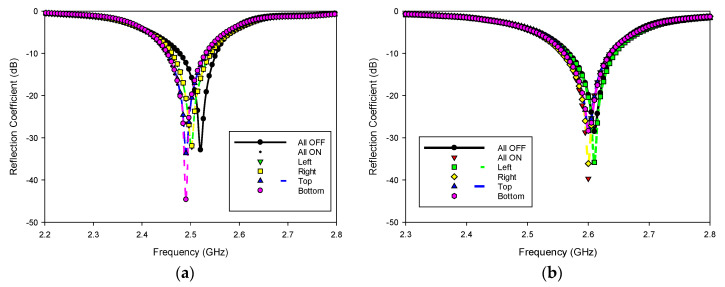
Reflection coefficient: (**a**) simulated; and (**b**) measured.

**Figure 9:**
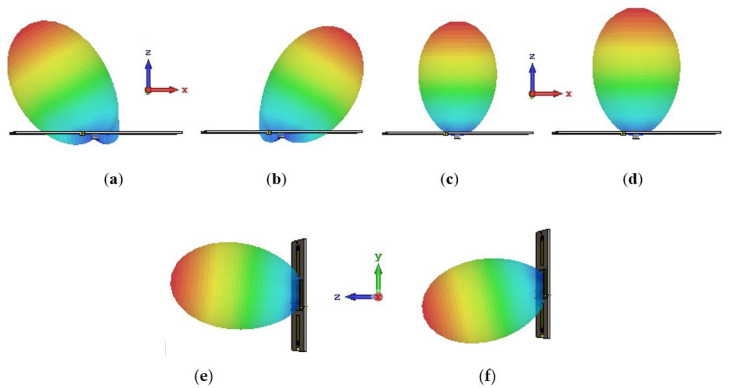
Simulated 3D beam direction on the broadside: (**a**) left; (**b**) right; (**c**) all OFF; (**d**) all ON; (**e**) top; and (**f**) bottom.

**Figure 10:**
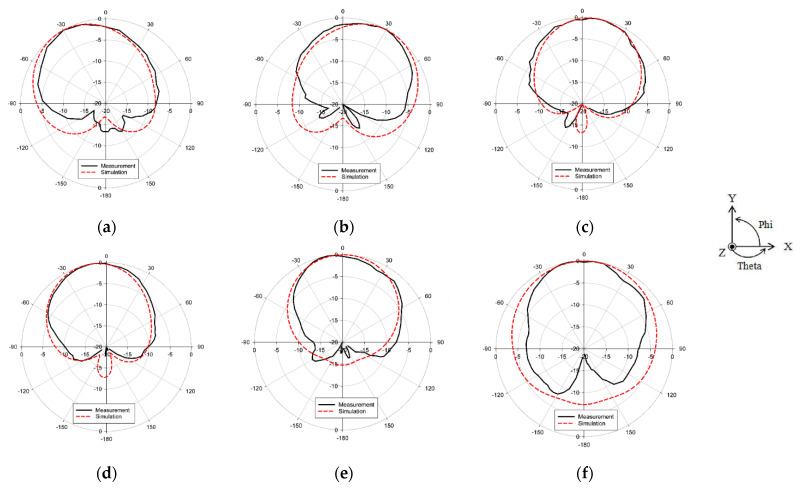
Normalized polar plot for measurement and simulation: (**a**) left; (**b**) right; (**c**) top; (**d**) bottom; (**e**) all OFF; and (**f**) all ON.

The result of the reflection coefficient for simulated and measured results is presented in Figure 8. It can be seen that all configurations have good matching and operate with minimum frequency shifts. The bandwidth is about 100 MHz (4% fractional bandwidth). However, the measured results are slightly shifted to a higher frequency. Figure 9 presents the simulated 3D radiation pattern, clearly showing the capability of the proposed antenna to tilt the beam into several targeted directions. Figure 10 illustrates the normalized radiation pattern (simulation and measurement) for all configurations. The simulated result obtained is 35° at the *x*-axis with gain of 1.7 dB and 9° and 15° at the *y*-axis with an average gain of 2.3 dB. A smaller tilting angle is produced for top-bottom configurations compared to left-right configurations due to the current being more coupled to the left and right parasitic elements due to the concentration of the current on the driven element. The result from this section validates the functionality of this antenna with a good beam-steering capability. Table 3 summarizes the comparison of steering angles for simulated and measured results for all configurations. Overall, good agreement is achieved between both the simulation and measurement results.

There are slight variations between the simulated and the measured results. Several reasons might contribute to these discrepancies. The variation might be mainly due to fabrication tolerance, especially since the parasitic dimension is very crucial for angle steering. In addition, efficiently soldering the PIN diodes in specific locations is a challenge in this design, as the PIN diode is inserted inside the substrate. Apart from that problem, the parasitic effects and insertion loss on the PIN diode slightly affect the antenna performance for the steering angle.

In addition, this design uses the conventional method of a reconfigurable antenna, in which the RF switches are mounted on the top or bottom layer of the parasitic elements. It could lead to a dimensional change in the antenna and slightly affect the antenna performance. Hence, to overcome this issue, the RF switches of the parasitic elements are proposed to be embedded inside the substrate. In this regard, it is highly competent to eliminate the intermodulation effects generated by the RF PIN diodes and the other passive elements associated with the PIN diodes.

Since the proposed design has successfully achieved targeted tilting angles in four different directions, this work is compared to previously reported antennas using the FR-4 substrate to develop the beam tilting reconfigurability, as listed in Table 4. It is clear that the angle tilting, tilt direction, and switch number of the proposed antenna feature comprehensive advantages. Moreover, the proposed antenna has among the largest scanning angles with less complexity, and it is easy to fabricate because of its single-layer configuration.

## 4. Conclusions

This paper presents a radiation pattern reconfigurable antenna with four tilting directions on a high-loss FR4 substrate. The design used a rectangular patch as the driven element and four parasitic elements located next to the driven element. The design begins with the strips of copper to be used as artificial switches for proof of concept on the full ground plane and continues with minor modifications made for employment of the real switches (PIN diode) and switching networks. The ground plane has been optimized to achieve a better steering angle. Depending on the switching configuration, the beam of the antenna can be steered in four directions: left, right, top, and bottom. The measured results demonstrate that the angle tilting has successfully achieved more than 30° with the left and right configurations. It only required four switches to attain the above aims. The proposed antenna shows the potential to serve as a base station in energy-efficient WSN applications.

## Figures and Tables

**Table 1:** Switching configurations of the proposed antenna.

Configurations	Tilt Direction	Switch 1	Switch 2	Switch 3	Switch 4
I	Left	OFF	ON	ON	ON
II	Right	ON	OFF	ON	ON
III	Top	ON	ON	OFF	ON
IV	Bottom	ON	ON	ON	OFF
V	Middle	OFF	OFF	OFF	OFF
VI	Middle	ON	ON	ON	ON

**Table 2:** Angle tilting comparison for different types of ground planes (simulated).

Type of Ground	Degree of Tilt Angle					
	Left	Right	Top	Bottom	All Off	All On
Full Ground	19°	19°	15°	18°	0°	0°
With DGP	30°	30°	10°	18°	0°	0°
Optimized DGP	32°	32°	9°	15°	0°	0°

**Table 3:** Summary comparison between the simulated and measured results of the tilting angle for all switch configurations.

Steering Angle	Left	Right	Top	Bottom	All OFF	All ON
Simulated	35°	35°	9°	15°	0°	0°
Measured	32°	32°	10°	17°	3°	2°

**Table 4:** Summary comparison with previous works on beam tilting antennas using FR-4 substrate.

Ref.	Technique/Structure	No. of Switches	Type of Switches	No. of Tilt Directions	Angle Tilting (°)	Electrical Size (λ_o_)^2^	Fractional Bandwidth (%)	Complexity
[13]	Four-Parasitic Array, Multilayer	4	PIN Diode	5	0, 13, 15, 10,12	1.67 × 1.83	1.7	Average
[15]	EBG and Slotted Antenna, Multilayer	14	PIN Diode	3	0, 25, 23 at 2.4 GHz 0, 28, 20 at 5.8 GHz	1.95 × 1.95	2.46 at 2.4 GHz 1.38 at 5.8 GHz	High
[16]	Metamaterial Inspired, Multilayer	4	PIN Diode	3	0, ±35	0.52 × 0.52	21.6	High
[17]	Antenna Array	16	PIN Diode	5	0, ±26, ±36	1.84 × 2.02	5.4	High
[18]	Slot and monopole	4	PIN Diode	3	35, −50, Omni	0.23 × 0.16	4.4	High
[19]	Cubical Array	4	CG2179M2	4	Sector at 0, 90, 180, and 270	1.69 × 1.69 × 1.69 (cube shape)	4.1	High
This paper	Four-Parasitic Array, Single Layer	4	PIN Diode	5	0, ±35, 10, 17	1.67 × 2.5	4	Average

## Data Availability

Not applicable.
